# Supplementary material for: Quantitative evaluation model of variable diagnosis for chest X-ray images using deep learning
Source: PLOS Digit Health. 2024 Mar 15;3(3):e0000460. doi: 10.1371/journal.pdig.0000460 (PMC10942047; doi:10.1371/journal.pdig.0000460)
Supplement: Attachment — Submitted filename: Answer_to_the_reviewers.20231023.docx [file pdig.0000460.s001.docx]

Answer to the reviewers

*Reviewer #1: This study presents a deep-learning model that quantitatively assesses chest X-ray findings with inter-clinician varying annotations. The authors implemented five different deep-learning models from 4-8 years ago to detect pleural thickening (PT) and scoliosis (S) in chest X-ray scans. For their analysis, they used an X-ray dataset containing over 82k chest X-ray scans labeled positive or negative with respect to PT and S. Two annotators re-examined 1k of the scans (independently). While I definitely understand the potential of such a study as a starting point for AI-based PT and S diagnosis, the manuscript feels a bit lacking with a “take-home message” as I’ll explain below. The manuscript is written and detailed very well and (almost) whenever I looked for some piece of information it was in the place I expected it to be so I could easily navigate. IMHO, this manuscript should be accepted upon bottom-line sharpening and better benchmarking.*

*1.1. What exactly is the final model suggested by the authors? It is unclear. Is it the (highest-scoring) efficient-net-based model? Why is the VAE so highlighted (across paper and in the abstract as well)? It underperformed all other models in almost every metric. Maybe I misunderstood something so it should be further elaborated…*

Our objective is to quantitate the probability of the physicians' findings by the output of the classification model and to validate this concept testing different models. We agree that highlighting VAE was somehow misleading and we did not aim to focus on certain model with the highest accuracy. In the present study, VAE was included in order to show our method is applicable even without pre-trained models. We revised the introduction section to explain the purpose of the study and the reason to compare those models (mainly in the second half of the introduction).

*1.2. Why did the authors use ResNet 50 and 101? Why not start with the simplest (i.e., 18)? It’s apparent that the ResNet-50 outperformed both 101 and the DenseNet-201 (and the VAE). Actually, I am not sure I was convinced that it’s not on-par with the efficient-net for the scoliosis learning task (any confidence intervals/significance evaluation for the difference? See [1] for example). I would have tried first a simpler approach to make sure it’s not an overkill.*

Actually, the same evaluation was conducted with ResNet-18, but its performance was about the same or slightly lower than ResNet-50. We added the result in the Table 1, 2, and the Fig 6. Indeed, the original purpose of evaluating these models was to show that the correlation between the model's predicted value and the probability of a doctor's annotation is a tendency that can be generally confirmed regardless of the model architecture or the number of parameters or the presence of prior training. We have shown the results of representative models.

*1.3. Benchmarking? Looks like the authors compared their models to none of the currently available methods, either generic, such as ConvNeXt [2] or medical-oriented such as REMEDIS [3], CheXzero [4], etc… It doesn’t have to be these ones but showing superiority against at least one state-of-the-art method is the minimum, o.w., what’s the point of the “new model”?*

Thank you for pointing it out. We added the evaluation on ConvNeXt. REMEDIS and CheXzero were not added because their learning methods were different and transfer learning using our data was difficult. Actually, the purpose of comparing models in this study is not to create a model that has higher classification accuracy than the golden standard, but rather to validate the idea that quantifying the probability of annotation is widely applicable regardless of the model architecture. We added paragraphs to the abstract and introduction section to explain the purpose of the study.

*1.4. The evaluation of research reproducibility poses challenges due to the absence of evaluation code or fine tuned weights for baselines or the method itself (GitHub link is broken). Also, the exact hyperparams used and the optimization methods were not detailed in the manuscript.*

We uploaded the fine-tuned models on the GitHub and added a description about optimization in the manuscript.

*1.5. What was the positives prevalence in the full dataset? Test set? Did you do a stratified CV? If so, you should have had around 10 positive cases (out of 1000) in the test set which makes it very hard to provide a meaningful evaluation. Did you try data augmentation?*

We added the number and ratio of positives in the Data Statistics subsection in the Result section. Actually, we did not do a stratified CV but randomly divided the training dataset so we corrected the explanation in the method section. There were 376.2 and 128.2 positives in each validation dataset in the average for pleural thickening and scoliosis, respectively. It was enough number for statistical evaluation and there were no significant biases in their occurrence.

Yes. We applied augmentation by random shift and random rotation to augment the input images in training (see “pre-processing” in the materials and methods section).

Minor comments:

*1.6. Add ref “In previous studies, the prevalence of idiopathic scoliosis in adolescents was estimated at 1 to 4%”*

Thank you for pointing it out. We added the citation.

*1.7 “nine-tenth” -> “nine tenths”, “annottation”, “is by” ?, Fig. 2 “Dence“*

Thank you for pointing them out. They have been corrected.

*1.8 Fig. 3. I would add a boundary box to refer to the zoomed area*

Thank you for the very good suggestion. We edited the image to add the boundary box.

*1.9 Fig. 5. The correlation of PP is qualitatively apparent (and expected) but for PN and NN it’s unclear. I would expect higher correlation for the NNs (compared to PNs) assuming that if both annotators reached the same conclusion (an in PP), why would the models not? It’s hard to compare without numbers/zoom-in to the relevant region. I suggest plotting each label in a different plot and providing the R^2.*

Thank you for the valuable suggestion. We tried the separate plots; however, it looked less informative since the number of PN was too small in the case of scoliosis. Instead, we added Table 3 to show separate averages and standard deviations of the correlation coefficients across 45 pairs for PP PN NN. The reason why the correlation was low for images with no findings (NN) is considered to be that the distribution of the output is saturated and asymmetrical around 0 as you pointed out.

*1.10 Fig. 6 “λp and λp” -> “λp and λs”*

Thank you for pointing it out. We corrected it.

*1.11. What were the (separate) mean R^2s across all 45 pairs of models for PP PN NN?*

We added the table of separate averages of the correlation coefficients across 45 pairs for PP PN NN (Table 3).

*1.12. Not sure I understand this sentence: “scoliosis was more clearly separated in the feature space so that the variance in output values was much larger than that of pleural thickening.” shouldn’t it be the other way around? If it’s easily separable the model’s variance should be lower…*

We meant that in the case of scoliosis, features were more easily observed so that the differences in the output values for individual images were much larger. We corrected the sentence to clarify this point.

*1.13. “Tables 1 and 2 summarize the performance of the models” -> “Tables 1 and 2 summarize the mean performance of the 10 cross-validated models”*

Thank you for pointing it out. We revised the text.

*1.14. AUC is defined for the first time in the discussion but used a few times before that.*

Thank you for pointing it out. We added the definition before its first use.

*1.15. What’s the re-checkout? Please define new terminology you use.*

We defined the "recheck" dataset in the materials and methods section and rephrased it.

*1.16. Feels like it should go to the intro and be more concise in the discussion (1-2 sentences): “Pleural thickening is a common finding on routine chest X-rays and its prevalence ranges from 1.8% in teenagers to 9.8% in adults aged 60 years and older. More than 90% of pulmonary thickening cases involve the apex of the lung, which is referred to as the ‘pulmonary apical cap’ and is generally recognized as a non-specific fibrotic change. Because the apical cap is an irregular density at the apex and is less than 5 mm in width, its annotation can be discordant among physicians and frequently ignored when interpreting chest X-rays for health screening. However, pulmonary thickening can be a manifestation of respiratory diseases such as mycobacterial infection and interstitial pneumonia”*

Thank you for pointing it out. We moved the paragraph into the introduction section and revised the discussion.

*P.S., please add line numbers in future submissions. it makes commenting and following-up very inconvenient.*

We are sorry for the inconvenience. We added the line numbers. And we are sorry that there still remains some confusion in the marked-up document which was converted from PDF to MS Word, but we couldn't fixed it when we used the numbered manuscript.

*Reviewer #2: I want to express my sincere appreciation for the opportunity to review the manuscript titled “Quantitative evaluation model of variable diagnosis for chest X-ray images using deep learning”. Having carefully examined the research, I would like to convey my interest and appreciation for the results presented in this study. The authors' work in shedding light on uncertainty of the deep learning models in diagnosis of chest X-ray images. The methodology employed was sound, and the analysis of the data yielded insightful conclusions. I would like to acknowledge the researchers for their effort. While the manuscript is indeed promising, I would like to offer a few notes for potential improvement. They are divided in major and minor notes:*

*Major*

*2.1. Authors mentioned in the discussion developing several models with different architectures followed by evaluating the posterior probability of the findings. However, within the limitations of my understanding, they only evaluated the VQ-VAE model.*

Actually, we evaluated the all models mentioned in the materials and methods section though we showed only the results of EfficientNet and VQ-VAE in the manuscript. We added the evaluations for the other models (Table 4).

*2.2. What is the benefit from building multitarget model compared to separate model for each diagnosis.*

In general, it improves learning efficiency when building predictive models for target variables that share some features.

*Minor*

*2.1. It would be helpful to mention the other transfer learning models developed in the abstract as the best model was one of them. More effort in adding details about the method and results should be done to provide readers with more details about the methodology used and the promising results.*

We revised that abstract to explain that our method can be applicable to both transfer learning and newly trained models.

*2.2. More citations must be included in key sentences in the introduction and discussion.*

We added some citations to support the sentences.

*2.3. Authors mentioned in the introduction that pleural thickening and scoliosis to be the most common findings in chest x-ray datasets followed by prevalence of the two findings. It’s recommended to added references that supports the first claim and add the references [21] and [35] to the paragraph as the pleural thickening and scoliosis prevalence was taken from them. They weren’t cited before this sentence.*

Thank you for the constructive comment. We revised the introduction section and added some citations.

2.4. In the introduction the pleural thickening and scoliosis were both mentioned to be the most prevalent in the population while reference [21] used only supports pleural thickening.

Following the suggestion, we revised the introduction section and added some citations.

*2.5. In the introduction try to mention the objective of the study in the last paragraph. It is advised that any justification for your objectives is mentioned before the objectives. Moreover, the objectives of the authors study should contain less of the methodology.*

Thank you for the valuable comment. We reorganized the introduction section to clarify the objective of the study.

*2.6. According to ethical considerations, authors should provide details about the IRB ethical approval number.*

This study was approved by the Institutional Review Board of the University of Tokyo (#19–324) and that of Nara Institute of Science and Technology (#2018-M-1). These descriptions are added in the revised manuscript (in Dataset subsection in the Materials and Methods).

*2.7. In the results the authors reported the average of correlation coefficient for the 45 pairs of models. It would be more informative to report the standard deviation to acknowledge the distribution of the values.*

We added a table of separate averages and standard deviations of the correlation coefficients across 45 pairs for PP PN NN (Table 3).

*2.8. The interpretation for lower accuracy in detecting pleural thickening is duplicated in the results and discussion. A more scholarly approach would involve addressing thvis matter exclusively within the discussion segment, coupled with the recommend solutions.*

Thank you for pointing it out. We revised the results and discussion sections according to the reviewer’s suggestion.

*2.9. More on table 1 and 2 results should be presented and discussed in the results and discussion sections. How different models architectures contributed to their performance? Is there any studies that used them and have a matching results?*

We appended the following paragraphs in the discussion.

According to the performance comparison of transfer learning models listed in Tables 1 and 2, models with a large number of parameters do not necessarily give better results. Similar trends can be seen in other previous studies using transfer learning. Unlike learning using a large number of image samples, fine-tuning using a limited number of samples, it is thought that learning efficiency will decrease if the number of parameters is too large. In particular, when performing transfer learning on images that are in a different domain from general photographs, such as medical images, care must be taken to use a smaller model to the extent that sufficient performance can be obtained.

*2.10. Comparison and discussion of the findings in this research with other studies is advisable. It would be interesting how the results of this study can be compared to other AI models developed for diagnosis of X-ray images.*

Thank you for the constructive advice. By comparing the evaluation method proposed in this study with other studies that evaluate model uncertainty, it would be possible to more accurately quantify the uncertainty inherent in the labels themselves annotated on X-ray images. We added this in Discussion section.

*2.11. Language editing for the manuscript would provide clearer presentation for the readers.*

We submitted the revised manuscript to an English proofreading.

*In conclusion, I believe that this manuscript has the potential to make a valuable contribution to the scientific community. The authors' commitment to rigorous research and their ability to generate thought-provoking results are truly commendable. I am looking forward to seeing how the authors address the points raised and further refine their work.*

*Reviewer #3:*

*3.1.	Mention the achieved accuracy in the abstraction.*

Our purpose is to propose a method to quantitate the probability of the physicians' findings by the output of the classification model. We added a more detailed explanation in the abstract and mention about the correlation between the output and the probability physicians' findings.

*3.2.	The previous research work is mentioned in the introduction section please create a separate section with the heading Literature Survey.*

Thank you for the suggestion. Because the introduced section in this paper is composed of several different topics, we gave up on making the literature survey into an independent section as it would be rather complicated, but we have organized the structure of the introduction and added references.

*3.3.	Add more literature citations in support of the literature survey.*

Thank you for the suggestion. We added 12 citations to refer to previous research works.

*3.4.	Cross-verify the figure citation (It is not Figure 1 replace it with Figure 1.)*

We exchanged the position of the Figures to correct citations and revised the manuscript accordingly.

*3.5.	Label Figure 3 as Figure 3a, 3b, and 3c instead of label upper and lower.*

Thank you for the constructive suggestion. We added labels for each subfigure.

*3.6.	State the limitation of your model.*

Thank you for the important comment. The data set used for the analysis was derived from Japanese participants and more than half were aged 20 to 30 years. It will be necessary to analyze a wider range of datasets in order to generalize the prediction models evaluated in our study. It should be also noted that probabilistic evaluations proposed in this approach is not simply applicable for the findings that are directly linked to critical diagnosis such as pneumonia, lung cancer, etc. We added these descriptions in the discussion section.

3.7.	*The data collection process is explained in a good style of writing.*

Thank you for your careful reading. We did our best to provide understandable explanations.

*Reviewer #4: Quantitative evaluation model of variable diagnosis for chest X-ray images using deep learning*

*The author(s) proposed a deep learning-based approach using vector-quantized*

*variational autoencoder to quantitative assess the probability*

*of detecting pleural thickening and scoliosis.*

*The author(s) “assumed the annotator's binary findings, that is, the presence or absence depends on the degree of radiological changes, and evaluated the probability of making an annotation.” What about intra-inter-observer variation, which has been well-established in the literature? For example, one annotator can make different findings depending on how they feel on the day. Also, two annotators can come up with two findings given the same X-ray image.*

*4.1.	The researcher(s) used two physicians to independently assess the Chest X-rays. How does this account for inter-intra-observer variability?*

We assumed that a physician's decision of making an annotation of pleural thickening or scoliosis is a Bernoulli process. It includes the randomness of intra-observer variability. Though the probability may depend on both the certainty of the finding and the physician's expertise, we found no significant difference between the two physicians. So, in this study, we ignored inter-observer variability, and assumed that this probability depends only on the image features. We added this description in Data statistics section in Resuts.

*4.2.	One of the biggest challenges with X-ray is that it is 2D, thus only giving one projection of the organ being imaged. As a result, an X-ray image can lead to an inconclusive diagnosis. It will be helpful if one can generate a 3D volume from one or multiple X-ray images.*

We agree that it is a very good point of view. Actually, there are some studies in that direction [1]; however, that is far beyond the extent of this study.

[1] Maken, P., Gupta, A. 2D-to-3D: A Review for Computational 3D Image Reconstruction from X-ray Images. *Arch Computat Methods Eng* 30, 85–114 (2023). https://doi.org/10.1007/s11831-022-09790-z

*4.3	It would be beneficial to briefly introduce the concept of uncertainty in medical diagnosis and its relevance, providing more context for readers unfamiliar with this concept.*

We added the following paragraph in the introduction section.

In the context of diagnostic support using machine learning, it is imperative to address the issue of uncertainty assessment. Uncertainty in diagnostic support can be broadly categorized into two types: Epistemic Uncertainty and Aleatoric Uncertainty. Epistemic Uncertainty stems from factors such as insufficient data or overly simplistic predictive models utilized in the diagnostic support process. Aleatoric Uncertainty, on the other hand, arises from fluctuations in patients' symptoms or noise inherent in the observations that serve as the training data.

In the development of diagnostic support systems, there are two primary objectives related to uncertainty assessment. One objective is to quantitatively characterize the statistical fluctuations inherent in the predictive models themselves, while the other is to evaluate the uncertainty associated with the labels provided by human observers, which serve as the ground truth for model training.

*4.4.	The author(s) assume physician diagnosis follows a normal distribution. Is this the same for both random and systematic misdiagnosis?*

We assumed that a physician's decision to make an annotation of pleural thickening or scoliosis is a Bernoulli process. It includes the randomness of intra-observer variability. It can be assumed that the probability depends on features of images, i.e., obviousness or difficulty of findings. On the other hand, systematic misdiagnosis will add some bias to the distribution of these probabilities. For example, if we can apply our method to datasets from different organizations, we can quantify and compare the difference in systematic bias between these systems.

*4.5.	The author(s) clipped the original chest X-ray images by 3000 x 3000 pixels and resized them into 256 x 256 pixels before input to the model. Why these values, it there something significant about these values? What happens if I choose different values?*

Since multiple devices were used in the annual health check, images of different sizes were included in the original dataset. We clipped 3000 x 3000 pixels as the largest area that can be shared by all images. We chose a multiplier of 2 to fit the general convolution model easily, but the size can vary depending on the design of the model. The larger input will provide more detailed information but will cost more computation (and require larger cache memory on GPU). We revised the discussion about resolution in Discussion.

*4.6.	Propose the author(s) re-arrange the model methodology and results section to be more structured. Start with Data, pre-processing (data augmentation), model training, Model evaluation and uncertainty.*

Thank you for pointing it out. We re-arranged the method section.

*4.7.	The saliency maps in the results are not mentioned in the methodology. Where do they come from, and how and why are there being used? This information should be in the methodology section.*

Deep-learning-based image analysis models are often regarded as quasi "black boxes", since even for their developers it often remains unclear why the algorithm delivers a certain result. We added an explanation in the method section.

*4.8	Has the author(s) validated their method with an external dataset?*

No. We could not find an appropriate dataset that is large enough and has multiple annotations for each finding to evaluate the probability of findings.

*Minor (but important)*

*4.9.	On page 5. The sentence: “Four models are transfer learning using pre-trained convolutional neural networks, and another model is a de novo model trained as an autoencoder from scratch” needs to be rephrased to become more understandable.*

Following the reviewer’s suggestion, we rephrased the sentence.

*4.10.	The authors should label each figure. For example Figure 4. It will be nice to have Figure 4 a) and b). Same with Figures 5, 6 and 7.*

Thank you for pointing out. We added labels for the subfigure.

--------------------
