# Supplementary material for: Quantitative evaluation model of variable diagnosis for chest X-ray images using deep learning
Source: PLOS Digit Health. 2024 Mar 15;3(3):e0000460. doi: 10.1371/journal.pdig.0000460 (PMC10942047; doi:10.1371/journal.pdig.0000460)
Supplement: Attachment — Submitted filename: Answer_to_the_reviewers.20240126.docx [file pdig.0000460.s002.docx]

Answers to the reviewers,

Reviewer #1: Thanks to the authors for the detailed and organized revision. The aim of this paper is now sharpened and its purpose is clearer.

Minor:

The GitHub repo is still unavailable. Maybe it’s set to private? I tried to look in the user’s page but there is no mention of this project.

It was set to private and now we changed it to public. We are deeply sorry that we misconfigured the project.

“One-tenth of the samples (Fig. 2) The Adaptive” missing period after fig 2.

Thank you for pointing it out. We revised the text.

Nitpicking:

“the proposed method was applied using multiple deep learning models with different architectures and a de novo model based on an autoencoder without

transfer learning.” -> “the proposed method was applied using multiple pre-trained and randomly initialised deep learning models with different architectures.”

We revised the paragraph as follows, to make the explanation clearer,

"To demonstrate that the proposed methodology is not dependent on the specific characteristics of any particular model, this study evaluates the approach across seven deep learning with different architectures. Six of these models are pre-trained on general photographic images, while one is a network that was randomly initialized and then trained as an autoencoder using chest X-ray images."

“1.0 × 10−4” -> “10−4”

Thank you for pointing it out. We revised the text.

I’d suggest shortening the abstract to start from “the purpose of this study..”. The intro before is too long for an abstract but that’s a personal taste.

We revised the whole abstract to shorten and remove the general background at the beginning to make the target of this study clearer.

It’s clear that the legends of Fig. 6 and 7 are duplicated. For the reader’s convenience, I’d consolidated the 12 panels of Figs 6 and 7 into one Fig with 2 panels- PT and S. each panel will show the performance of all the tested models (hued by model)

Thank you for the suggestion. We gathered the panels in Fig. 6 and 7 into one figure with two panels, showing the average and standard deviation of all 6 models. And also, changed Fig. 8 using the same style.

Reviewer #2: The github link is still broken

It was set to private and now we changed it to public. We are deeply sorry that we misconfigured the project.

Reviewer #4: The authors have addressed my comments

Thank you for reviewing our papers.
